# Report of the Work of the past Quarter

**Published:** 1919-06-07

**Authors:** 


### THE LONDON HOSPITAL.

### Report of the Work of the Past Quarter.

THE Committee and the medical and surgical staff of the hospital have been in the closest co-operation in their attempts to inaugurate the system of medical and surgical units, referred to in the last quarterly report. They are now awaiting a definite statement from the Board of Education as to what sum will be allotted to the hospital for the carrying out of the teaching work. Government departments of necessity move slowly, and although it is known that the Board of Education is very sympathetic with the scheme, a written statement of the condition on which they will make a grant and the exact amount of the grant has not yet been received. Without this written statement it has not been possible to inform suitable applicants for the post exactly what the conditions of their service will be, and what is the length of tenure of office. Obviously a surgeon or physician who may find this kind of work congenial cannot undertake it and give up all private practice unless he is given a definite promise that the appointment will hold good for a certain term of years; and an endeavour is being made to arrange this matter. A unit for the study of women's diseases, and especially midwifery, with an ante-natal and post-natal department attached to it, will probably be inaugurated; also a unit for the study of skin diseases and syphilis.

### NURSES' SALARIES.

At the last Quarterly Court the Chairman informed the Court that the question of nurses' salaries would again have to be reconsidered. This has been done, and the following salaries have been fixed :—A probationer in her first year is to receive £20; in her second year, £25; ward nurses, £40 a year; private staff nurses from £45, rising up to £70 a year; ward sisters from £50 to £90 per annum; and there are a few special appointments, heads of departments, and so on, from £65 to £110 and over. Pensions for nurses, after twenty years' service, are fixed at from £60 to £110 per annum. A tutor-sister, who will organise the whole scheme for the training of the nursing staff, has been appointed.

### THE TREATMENT OF PENSIONERS.

It was originally decided not to treat pensioners at the hospital, not of course because the Committee were not anxious that they should be treated, but because they felt that the Government ought to make other arrangements and not occupy the time or the beds at a voluntary civilian hospital. The hospital has been asked to reconsider this decision, and having been informed that there are 8,000 pensioners in Stepney alone, it does not seem right to the Committee that the experience and equipment associated with this great hospital should be debarred from these men. The Committee are in communication with the honorary staff concerned.

### INSURANCE OF THE HOSPITAL AND ITS CONTENTS.

Arrangements for the insurance of the hospital for a period of seven years, together with its contents, have just been made. The hospital buildings and contents have been valued at £820,850, and the insurance for a seven-year policy amounted to £2,776 18s. 8d.

### DEATHS.

The Committee report with much regret the death of Mr. Rowland Plumbe, the consulting architect of the hospital, who has been a member of the House Committee for thirteen years. Mr. Rowland Plumbe was responsible for the rebuilding of the hospital which has taken place during the last twenty years. The Committee also announce with great regret the death of Dr. F. J. Smith, one of the most well-known and most popular physicians of the hospital. A special memorial service was held in the hospital church, and Dr. Robert Hutchison made a funeral oration. The Committee also regret to announce the death of Sir Victor Buxton, who died as the result of a motor accident last week. He had been a Life Governor since 1865.

### IMPORTANT GIFTS.

An anonymous donor has most generously made a present to the hospital by sending a cheque for the purchase of a house costing £1,550, to be used as a hostel for pregnant women who are undergoing treatment at the hospital. Unfortunately a house which was in view has been sold by private treaty, but suitable premises are being searched for. Sir Frederick Green, K.B.E., vice-president, has sent £1,000 to the hospital. A valuable gift of ether, etc., has been received from the American Red Cross, and other valuable gifts from the British Red Cross Society. The Committee report with very great pleasure the gift of £15,000 from the Goldsmiths Company, to found a chair at this hospital for bacteriology.

### THE WAITING LIST.

The hospital is still working at very great pressure, a fact which will be appreciated when it is learned that the number of cases waiting their turn for admission is between 500 and 600. Patients are written for to come in on the day that a patient is due to leave, and except for a few beds which have to be kept vacant for emergencies, beds are practically never empty. Every hospital has these waiting lists, and it shows that the beds provided for the sick poor are totally inadequate, a fact which will have to be taken into account by the Ministry of Health.

